# Progress in Surgery

**Published:** 1898-01-29

**Authors:** 


### PROGRESS IN SURGERY.

### SURGERY OF THE BRAIN.

**Epilepsy.**—Among recently-published cases of operative interference for Jacksonian epilepsy the following are of interest. Summers[1] reports the case of a young man, aged 20, who, four years previous to coming under his care, sustained a compound depressed fracture of his skull, involving the frontal and left parietal bones. A year later the depressed bone was elevated, but some paralysis still remained, and soon after epileptiform seizures began, and gradually became very severe. On examination it was found that the elevated bone had sunk again, and was causing paresis and atrophy of the right limbs and some interference with speech in addition to the epileptic attacks. A second operation, which consisted in removing all depressed bone and inserting a piece of gold foil between the dura and the scalp, was followed by improvement of all symptoms, and for the last eighteen months there have been no fits. The author does not yet claim the case as a cure, but fairly considers the result encouraging. He has operated for similar conditions in four other cases, in two with no improvement, in the other two with great benefit if not actual cure. He is a strong advocate for elevation of all depressed fractures at the time of accident, whether accompanied by symptoms or not, as a preventive measure. He further expresses his opinion against operation in cases of Jacksonian epilepsy in which no distinct history of trauma is forthcoming.

Beach[2] relates the case of a girl whose epilepsy began eight years after a compound comminuted fracture. For fifteen months after an operation had been performed she remained free of fits, till she was again injured by a fall, when they returned. On opening up the old wound it was found that the brain and the bone were adherent. Gold foil was introduced to prevent this re-forming, and since this was done, five years ago, the patient has only had occasional fits, mostly associated with menstrual disturbances. The case reported by Philip James, Wellington, N.Z..,[3] is of double interest, first, on account of the success attending the use of a silver plate to prevent adhesions, and also from the light it throws on some points in localisation. It refers to a man aged 24, who 11 years previous to the onset of fits sustained a severe injury to his head. His fits were associated with visual disturbances, and it was found that the tender spot in the cicatrix corresponded to the position of the angular gyrus. Pressure on this spot produced the subjective visual phenomena which preceded an attack, namely, dimness of sight progressing to absolute darkness; at the same time he trembled all over, and the heart beat forcibly. The operation consisted in exploring the cicatrix, freeing adhesions, and inserting a plate of silver. No fits have since occurred, the visual disturbances have ceased, and the silver plate has caused no discomfort.

Stewart, of Leith,[4] reports a most interesting case of epileptiform convulsions also associated with visual phenomena in the form of amblyopia and complete left homonymous hemianopsia and hemiacromatopsia, with concentric contraction of the healthy field. The patient was 24 years of age, and it had been observed that the right side of his head was somewhat larger than the other. There was tenderness or percussion over the region of the posterior fontanelle. The skull was trephined over the right occipital lobe, and the exposed cortex was found to be not thicker than ordinary blotting-paper. On incising it and introducing the finger, it was found to enter an enormously dilated lateral ventricle, from which cerebro-spinal fluid freely escaped. This continued to escape for some days, but eventually stopped, and the patient remained free of fits for six months, when they returned. A small drainage-tube was inserted through the wound, and the fits again ceased so long as the drainage was continued, but recurred occasionally afterwards. The patient was very much better after the operation, both as regards his fits and his vision, which all but became normal.

A case of hæmorrhage into the white substance of the brain, followed by aphasia, hemiparesis, and Jacksonian spasms, is recorded by Borsuk and Wizel.[5] After the removal of a clot from the dura, Jacksonian spasms persisted, and the brain substance was consequently explored by means of a long hypodermic needle, with the result that a quantity of blood-stained fluid was withdrawn. A free incision gave vent to broken-down brain substance and blood-stained fluid, and the wound, after draining for some time, healed up, leaving the patient free of his symptoms.

**Cerebral Localisation.**—Two cases in which certain visual symptoms were of value as localising guides for operative purposes have already been referred to: (1) James' case, in which dimness of sight gradually progressing till all was dark, associated with tenderness over the situation of the angular gyrus led to the freeing of adhesions in that locality, with relief to the symptoms. (2) Stewart's case, with left homonymous, hemianopsia, and hemiacromatopsia, and amblyopia, together with tenderness over the occipital lobe. In this case a congenitally dilated lateral ventricle (unilateral hydrocephalus) caused thinning of the cortex, and drainage was followed by great improvement. In the *Clinical Journal* for July, 1897, Stoddart gives a valuable summary of the localising significance of various clinical signs: (1) Mental symptoms are of little definite value. Loss of memory and moral deterioration are often associated with lesions of the frontal lobes. Mental symptoms like those of general paralysis often follow disease of the posterior third of the hemispheres. (2) Persistent headache is also a vague guide. A cerebellar tumour of one side is often associated with pain in the opposite frontal region. Pain in the nape of the neck suggests a sub-tentorial growth. (3) Cephalic tenderness, if only elicited on firm pressure or percussion, indicates a tumour of the meninges near the tender spot, but if superficial the tumour is in the brain substance. Tenderness over the face occurs with cerebellar growths. (4) Fits of the Jacksonian type are valuable guides to locality of lesions. The motor areas round the fissure of Rolando are well known. The character of the aura is often suggestive, *e.g*, seeing complicated pictures (angular gyrus); ringing of bells (superior temporo-sphenoidal); metallic taste (hippocampus major). (5) Cranial nerves: Loss of smell occurs when frontal tumours press on the olfactory lobes; also sometimes in cerebellar disease. Optic neuritis is most intense in occipital and cerebellar disease, but occurs in almost all kinds of intracranial mischief, and is therefore of little diagnostic value. Complete blindness often follows lesions of optic chiasma, or splenium of corpus callosum. Hemianopia follows lesions of optic chiasma and occipital lobe or intermediate parts. When the lesion is in the optic radiations hemianæsthesia accompanies the hemianopia; when in the occipital lobe it does not. When ophthalmoplegia of the opposite side accompanies hemianopia the lesion is near the external geniculate body, and causes pressure on the crus. Irregular forms of hemianopia are due to lesions of the chiasma. Third nerve: Paralysis of the muscles supplied by this nerve follow lesions of it. Fourth nerve

Homonymous diplopia. Sixth nerve: Convergent squint. Fifth nerve: Paresis of muscles of mastication, with loss of sensation of face and scalp in front of line joining the two ears. Loss of sensation in nasal cavities and mouth as far back as the tonsils and soft palate; loss of taste in front part of tongue. Jaw deviates to paralysed side if mouth opened widely. Seventh nerve: Paralysis of trunk causes inability to raise forehead, frown, close eye, or raise upper lip on that side. If cerebral lesion paralysis only noticeable in lower part of face by inequality of naso labial folds. If limited to cells of facial nucleus patient can still whistle through the orbicularis ovis receiving fibres from the hypoglossal. Eighth nerve: Deafness. Ninth nerve: Loss of sensation in pharynx and back of tongue. Twelfth nerve: Tongue paralysed, and deviates to paralysed side when protruded. No wasting of tongue when lesion is cerebral. Complete hemianæsthesia, especially if associated with hemianopia, means lesion of posterior end of internal capsule. Knee jerks are diminished or lost in irritative lesions of the cerebrum and paralytic lesions of the cerebellum, and are increased in paralytic lesions of the cerebrum and irritative lesions of the cerebellum. These statements are not absolute. Anæsthesia of little value. Corpus callosum: Unilateral fits associated with hemiplegia of opposite side, suggest a lesion of the corpus callosum. Lesions of lenticular nucleus may cause rise of temperature. Optic thalamus: Mobile spasm of one side, with hemiplegia in opposite side. Corpora quadrigemina: Ophthalmoplegia and staggering gait, with increased knee jerks. Crus cerebri: Paralysis of third nerve of one side, with hemiplegia of other side. Pons: Paralysis of fifth, sixth, seventh, and eighth nerves of one side, with paraplegia of other side. Medulla: Paralysis of ninth, tenth, eleventh, and twelfth nerves of one side, with hemiplegia of other side. Cerebellum: Lesion of anterior part of middle lobe causes patient to fall forward; of posterior part to fall backward. Nystagmus most marked on side of lesion. Weakness of back muscles. Lateral lobe: Rotation to side of lesion, superior peduncle: most marked on head and trunk; middle peduncle: most marked in trunk; inferior peduncle: rigid extension of limbs of same side, with flexion of opposite side. Knee jerk diminished on side of lesion.

[1] Med. Record, Ap. 24, 1897. [2] Boston Med. and Surg. Jour., March, 1897. [3] Australasian Med. Gaz, May, 1897. [4] Scottish Med. and Surg. Jour., Aug. 1897. [5] Arch für Klin. Chir., 1897. [6] Clin. Jour., July, 1897; and Summary Glas. Med. Jour., Sept., 1897.

